# Alu hypomethylation in ischemic stroke: Associations with selected clinical characteristics

**DOI:** 10.1371/journal.pone.0360371

**Published:** 2026-10-21

**Authors:** Doungkamol Siri-archawawat, Sirithip Chuaijit, Apiwat Mutirangura, Jerald Tan, Jirapan Thongsroy

**Affiliations:** 1 School of Medicine, Walailak University, Nakhon Si Thammarat, Thailand; 2 Walailak University Hospital, Walailak University, Nakhon Si Thammarat, Thailand; 3 Research Center in Tropical Pathobiology, Walailak University, Nakhon Si Thammarat, Thailand; 4 Center for Excellence in Molecular Genetics of Cancer and Human Diseases, Chulalongkorn University, Bangkok, Thailand; 5 Department of Anatomy, Faculty of Medicine, Chulalongkorn University, Bangkok, Thailand; 6 Department of Biochemistry, Yong Loo Lin-School of Medicine, National University of Singapore, Singapore, Singapore; Griffith University, AUSTRALIA

## Abstract

This study investigated Alu methylation levels in 85 ischemic stroke patients and 50 healthy controls, using Alu-Combined Bisulfite Restriction Analysis (COBRA) a quantitative approach to assess methylation. Ischemic stroke patients had significantly lower overall Alu methylation as compared to controls (P < 0.0001). Alu methylation was negatively correlated with age in both the control group (r = −0.6131, P < 0.0001) and the ischemic stroke group (r = −0.2834, P = 0.0086). Moreover, among age-matched ischemic stroke patients, there remained a difference between control and ischemic stroke patients (P = 0.0182). Alu methylation remained associated with ischemic stroke status after adjustment for age and sex (adjusted OR = 0.841, 95% CI = 0.709–0.979, P = 0.0327). Within the ischemic stroke group, disease duration was not significantly correlated with Alu methylation in the unadjusted analysis (r = −0.1594, P = 0.1450). However, after adjustment for age and sex, longer disease duration was associated with lower Alu methylation (β = −0.556, 95% CI = −0.994 to −0.117, P = 0.0137). Alu methylation was lower in patients with a history of multiple stroke events (P = 0.0323) and in those with Fazekas scores of 2–3 (P = 0.0324) in the unadjusted comparisons, but neither association remained statistically significant after adjustment for age and sex (P = 0.0542 and P = 0.0684, respectively). Stroke circulation location was not associated with Alu methylation. These findings support an association between Alu methylation and ischemic stroke status. Longer disease duration was also associated with lower Alu methylation after adjustment for age and sex. However, the cross-sectional design does not establish causality, disease progression, or predictive or prognostic value. Longitudinal studies are needed to determine the clinical relevance of these findings.

## Introduction

Stroke is one of the main diseases of the brain blood vessels, the defining feature of which is the acute cessation of blood supply to the brain, resulting in the loss of neurological function [1,2]. It ranks among the top causes of death and is one of the most important sources of long-lasting disability in people around the globe, thus stroke is a major health problem worldwide [3]. Every year, around 12 million individuals suffer a stroke, with 7 million fatalities and numerous others surviving but living with lifelong disabilities. According to the WHO, stroke was the second leading cause of death globally in 2021 [4].

Stroke is characterized as a multifactorial condition and associated with genetics and epigenetics [5–7]. Several studies have reported epigenetic changes and alterations in DNA methylation associated with stroke, and genomic instability is believed to contribute to its development [8–10]. Earlier findings showed that stroke patients have a lower level of DNA methylation compared to healthy individuals, and this is influenced by stroke progression [11,12].

DNA methylation has two primary functions that the regulation of gene expression and the prevention of genomic instability [13–17]. Alu methylation, which is a type of DNA methylation, occurs in short interspersed repetitive elements known as Alu elements. Alu elements account for over 1 million copies, up to 10% of the human genome [18]. Alu methylation helps prevent the accumulation of endogenous DNA damage, and oxidative stress [19], which may induce major factor contributing to stroke. Moreover, many studies have found that genomic hypomethylation could lead to genomic instability [20,21].

Ischemic stroke is diagnosed and classified using clinical assessment and neuroimaging [22–24]. However, the relationship between Alu methylation and ischemic stroke remains unclear. Therefore, this study aimed to compare Alu methylation levels between patients with ischemic stroke and healthy controls and to examine the associations between Alu methylation and selected clinical characteristics.

In this study, we used Alu-Combined Bisulfite Restriction Analysis (COBRA) to detect the levels of Alu methylation in all the samples. COBRA is a highly accurate method for quantitative and patterns methylation analysis of Alu interspersed repetitive sequence, whereas pyrosequencing cannot fully patterns of Alu methylation [25,26].

## Materials and methods

### Participants

This cross-sectional, single-center study was conducted at Walailak University Hospital. A priori sample-size calculation was performed for the comparison of two independent groups. Assuming a two-sided significance level of 0.05, 90% statistical power, and an expected effect size (Cohen’s d) of 0.68 estimated from the systolic blood pressure comparison in our previous study of Alu methylation in hypertension [27], at least 47 participants were required per group. The final analysis included 85 patients with ischemic stroke and 50 apparently healthy controls.

Adults aged 18 years or older were recruited at Walailak University Hospital from 1 October 2024 to 31 March 2025. The stroke group included patients diagnosed with ischemic stroke who were receiving care at the neurology clinic. Among the 90 patients screened, two were excluded because of current or previous cancer, and three declined to participate. Therefore, 85 patients were included in the final analysis. All patients in the stroke group had ischemic stroke; patients with hemorrhagic stroke were not included.

Fifty apparently healthy adults aged 18 years or older were recruited as controls. Their health status was assessed using medical history, routine health screening, and blood test results. Individuals with a known diagnosis of hypertension, dyslipidemia, diabetes mellitus, cardiovascular disease, cancer, or any other chronic disease were not enrolled as controls. Specific inflammatory biomarkers were not measured at the time of blood collection.

All procedures were conducted in accordance with relevant guidelines and regulations and were approved by the Ethics Clearance Committee on Human Rights Related to Research Involving Human Subjects at Walailak University, Nakhon Si Thammarat, Thailand (Ethical Project No. WU-EC-MD-5-311-66, approval period: 15/05/2024 to 14/05/2025). The recruitment period for this study was from 01/10/2024 to 31/03/2025 at Walailak University Hospital. Written informed consent was obtained from all participants prior to enrollment. Confidentiality of participant-identifiable information was strictly maintained throughout data collection.

### DNA extraction and bisulfite modification

Whole blood was centrifuged at 1,500 × g for 15 min at 4 °C to collect the buffy coat. Each buffy coat was mixed with 475 µL of lysis buffer II, 25 µL of 10% SDS, and 10 µL of proteinase K (20 mg/mL). Samples were vortexed and incubated overnight at 50 °C. Genomic DNA was extracted using phenol-chloroform-isoamyl alcohol (25:24:1), precipitated with ethanol, washed with 70% ethanol, and resuspended in 20 µL of H_2_O. DNA concentration was measured before conversion. For each sample, 750 ng of DNA was adjusted to 20 µL with H_2_O and bisulfite-converted using the EZ DNA Methylation-Gold Kit (Zymo Research, USA) according to the manufacturer’s instructions. The DNA was then purified and eluted in 10 µL of M-Elution Buffer [28].

### ALU-Combined Bisulfite Restriction Analysis (COBRA)

This method can identify the amount of methylation at CpG loci using a set of conserved primers for each IRS (interspersed repetitive sequence). One microliter of bisulfite-converted DNA from each sample was used for each COBRA-Alu PCR reaction; DNA concentration was not remeasured after conversion. The methylation level of Alu in samples was assessed using 1X PCR buffer (Qiagen, Germany), 0.2 mM deoxynucleotide triphosphate (dNTP) (Promega, USA), 1 mM magnesium chloride (Qiagen, Germany), 25 units of HotStarTaq DNA Polymerase (Qiagen, Germany), and 0.3 µM primer pair. With R = A or G and Y = C or T, we used ALU-Forward (5’-GGYGYGGTGGTTTAYGTTTGTAA-3’) and ALU-Reverse (5’-CTAACTTTTTATATTTTTAATAAAAACRAAATTTCACCA-3’). The following program was utilized for Alu amplification: 15 min of initial denaturation at 95 °C, 40 cycles of denaturation at 95 °C for 45 sec, annealing at 57 °C for 45 sec, extension at 72 °C for 45 sec, and a final extension at 72 °C for 7 min. Using 2 U of TaqI (Thermo Scientific, USA), 2 units of TasI (Thermo Scientific, USA), 5X NEB3 buffer (New England Biolabs, USA), and 1 µg/µl bovine serum albumin (BSA) (New England Biolabs, USA), COBRA was carried out on Alu PCR products. Each digestion reaction was incubated overnight at 65 °C. The digestion products were then separated on an 8% acrylamide gel and stained with SYBR (Lonza, USA). The intensity of the Alu methylation band was examined and evaluated using a Typhoon FLA 7000 imager [28].

### Methylation analysis

The COBRA results were separated into four groups: hypomethylation at both CpGs (uCuC), hypermethylation at both CpGs (mCmC), partial methylation at mCuC, and partial methylation at uCmC. The Alu methylation levels for each group were determined by measuring the intensity of COBRA-digested Alu products. The band intensity of Alu methylation was observed and measured using the Gel Doc XR+ imaging system and Image Lab software (Bio-Rad Laboratories, USA). Alu methylation analysis was performed using the band intensities of five Alu products with sizes of 133, 90, 75, 58, and 43 bp (Fig 1). Representative COBRA-Alu digestion products separated on an 8% polyacrylamide gel are shown in S1 Fig. The method for calculating ALU methylation was A = 133/133, B = 58/58, C = 75/75, D = 90/90, E = 43/43, and F = 32/32 or (E + B) – (C + D). Alu methylation levels were determined using the formula below: Alu methylation level percentage (%Alu methylation) = 100 x (2F + 2D + 2C)/(2A + 2C + 2D + 2F); mCmC loci percentage (%mCmC) = 100 x F/(A + C + D + F); uCmC loci percentage (%uCmC) = 100 x C/(A + C + D + F); mCuC loci percentage (%mCuC) = 100 x D/(A + C + D + F); and uCuC loci percentage (%uCuC) = 100 x A/(A + C + D + F) [29].

**Fig 1:**
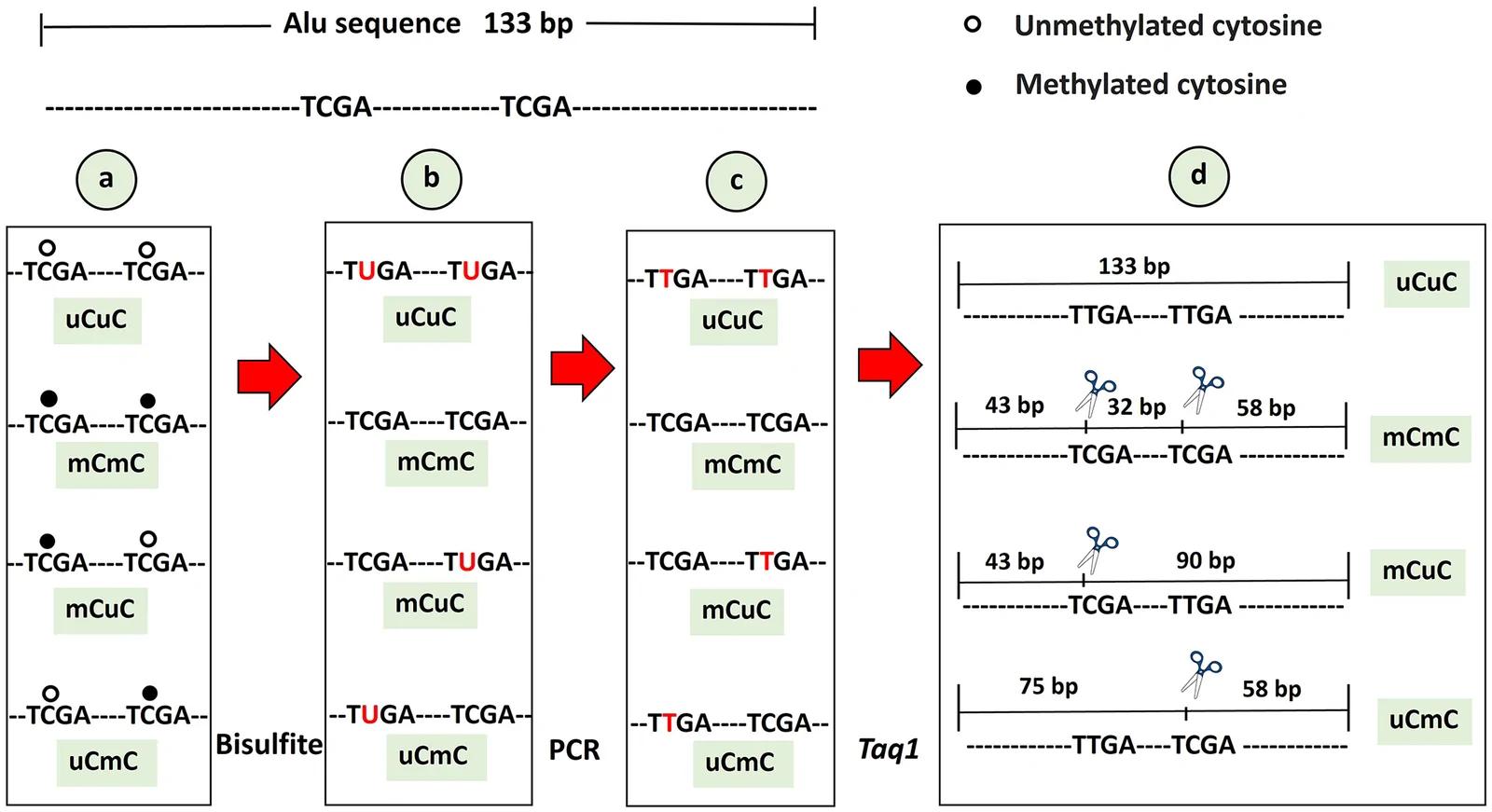
The Alu sequence contains 133 bp with two CpG dinucleotides. (a) A schematic representation of the COBRA-Alu assay illustrates the methylation patterns of the Alu sequence, including fully unmethylated loci (uCuC), fully methylated loci (mCmC), and two partially methylated forms (mCuC and uCmC). (b) During bisulfite treatment, methylated cytosine bases remain unchanged, whereas unmethylated cytosine bases are converted to uracil bases. (c) PCR products obtained from the COBRA-Alu methylation pattern assay. (d) The PCR products were digested with the TaqI restriction enzymes. The digested products are uCuC (133 bp), mCmC (43, 32, and 58 bp), mCuC (43 and 90 bp), and uCmC (75 and 58 bp).

### Statistical analyses

The normality of Alu methylation levels and methylation pattern percentages was assessed using the Shapiro-Wilk test. Normally distributed continuous data are presented as mean ± standard deviation (SD), whereas non-normally distributed data are presented as median and interquartile range (IQR). Categorical variables are presented as numbers and percentages. Because Alu methylation was not normally distributed and at least one group showed a non-normal distribution for each methylation pattern, comparisons between two independent groups were performed using the two-sided Mann-Whitney U test. The two-sided Wilcoxon matched-pairs signed-rank test was used to compare Alu methylation levels between the 16 age-matched pairs of controls and patients with ischemic stroke. The Mann-Whitney U test was also used to compare Alu methylation levels according to hypertension and diabetes mellitus status within the stroke group. Spearman’s rank correlation coefficient (r) was used to assess associations between Alu methylation levels and continuous variables.

Multivariable logistic regression was used to examine the association between Alu methylation and ischemic stroke status after adjustment for age and sex. Recorded comorbidities were not included in the logistic regression model because they were absent among controls and dyslipidemia was present in all patients with ischemic stroke, preventing reliable estimation of their independent effects. Within the stroke group, separate multiple linear regression models were used to examine associations between Alu methylation and disease duration (years), stroke event history (multiple versus single), Fazekas score category (2–3 versus 0–1), or stroke circulation location (posterior versus anterior), after adjustment for age and sex. In each linear regression model, Alu methylation was entered as the continuous dependent variable, while age, sex, and one clinical characteristic were entered as explanatory variables. Patients with missing Fazekas scores or involvement of both circulations were excluded from the respective analyses. For each linear regression model, the normality of the model residuals was assessed separately using the Shapiro-Wilk test. All tests were two-sided, and P < 0.05 was considered statistically significant. Analyses were performed using GraphPad Prism software.

## Results

### Alu methylation and pattern in stroke patients

The study included 50 healthy controls and 85 patients with ischemic stroke. No patients with hemorrhagic stroke were included. Participant characteristics are summarized in Table 1. Among the 85 patients with ischemic stroke, 67 (78.82%) had hypertension, all 85 (100%) had dyslipidemia, 30 (35.29%) had diabetes mellitus, 2 (2.35%) had atrial fibrillation, and 8 (9.41%) had coronary artery disease.

**Table 1:** Participant characteristics by study group.

	Group	
	Normal control	Ischemic stroke
*N*	50	85
Sex Male Female	10 (20.00%) 40 (80.00%)	63 (74.12%) 22 (25.88%)
Age (years) (mean±SD)	43.16 ± 15.40	64.63 ± 8.96
Comorbidities, n (%) Hypertension Dyslipidemia Diabetes mellitus Atrial fibrillation Coronary artery disease	0 (0.00%) 0 (0.00%) 0 (0.00%) 0 (0.00%) 0 (0.00%)	67 (78.82%) 85 (100.00%) 30 (35.29%) 2 (2.35%) 8 (9.41%)

Note: Controls with known chronic diseases were not eligible for inclusion.

In this study, we used the ALU-Combined Bisulfite Restriction Analysis (COBRA-Alu) method to measure the level of Alu methylation in all samples. Alu methylation values were not normally distributed in either the control group (Shapiro-Wilk W = 0.9415, P = 0.0155) or the ischemic stroke group (W = 0.9425, P = 0.0009). The findings exhibited a significant decrease in levels of Alu methylation in ischemic stroke patients compared to controls (P < 0.0001, Fig 2a). To confirm sex did not affect Alu methylation, we divided each group into male and female subgroups in both control and ischemic stroke patients. The sex comparisons showed no significant differences, confirming that sex does not impact Alu methylation levels in either normal control individuals or patients who had a ischemic stroke (Fig 2b).

**Fig 2:**
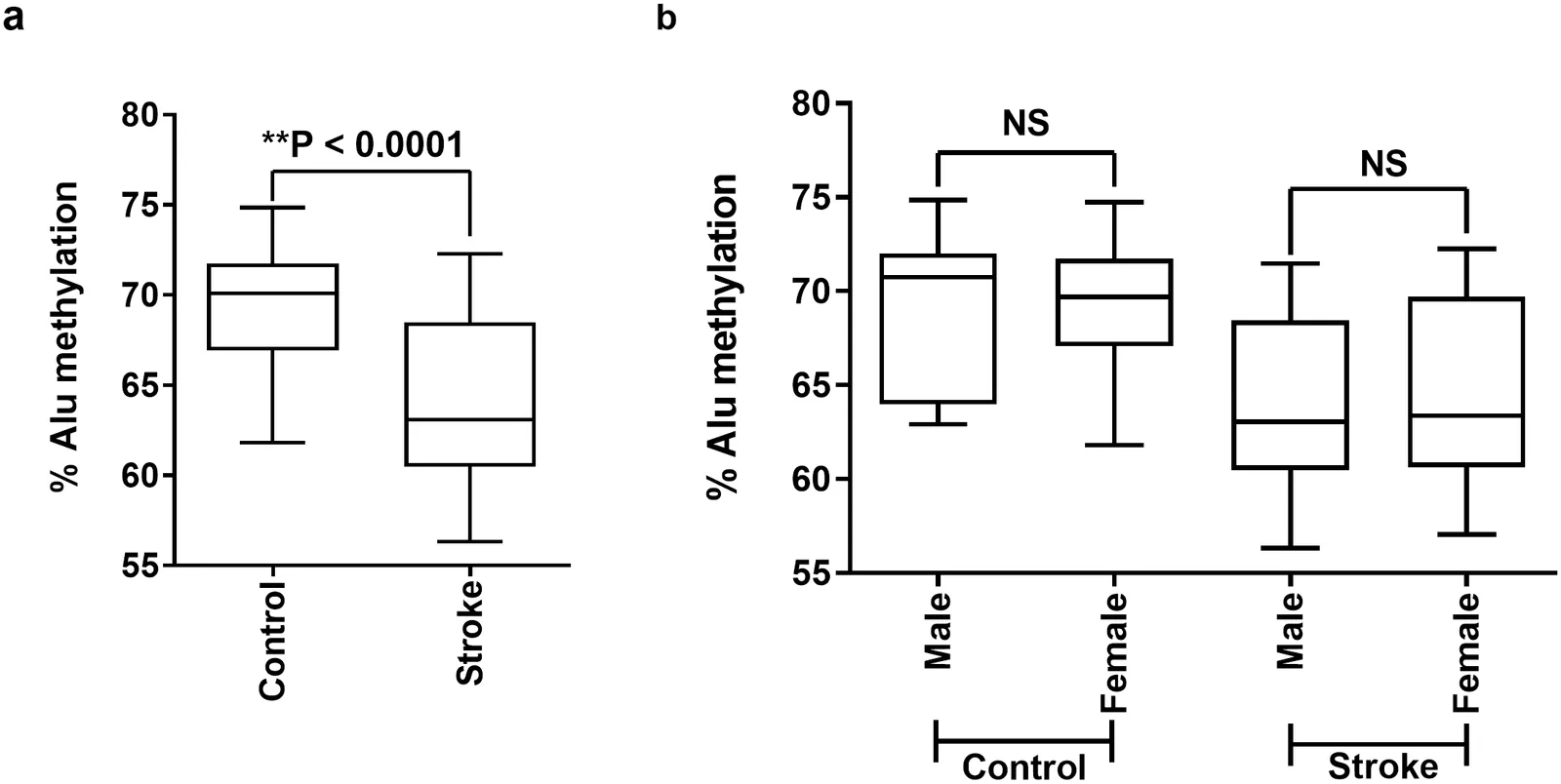
The Alu methylation levels in control and ischemic stroke patients. (a) The percentage of each Alu methylation level in control and ischemic stroke patients. (b) Comparison of Alu methylation levels between males and females in both control and ischemic stroke groups. The results are presented as box plots, where the boxes represent the interquartile range (25th to 75th percentile), the median lines represent the 50th percentile, and the whiskers represent the minimum and maximum values. **P < 0.01 (Mann-Whitney U test).

The pattern of Alu methylation was classified into four groups: fully unmethylated loci (uCuC), fully methylated loci (mCmC), and two partially methylated forms (mCuC and uCmC), as described in the methods section and Fig 1. In ischemic stroke patients, there was a significant decrease in mCmC (P = 0.0018) and a significant increase in uCuC and mCuC (P < 0.0001 and P = 0.0317, respectively) (Figs 3a-3c). However, uCmC showed no significant change in ischemic stroke patients compared with controls (Fig 3d).

**Fig 3:**
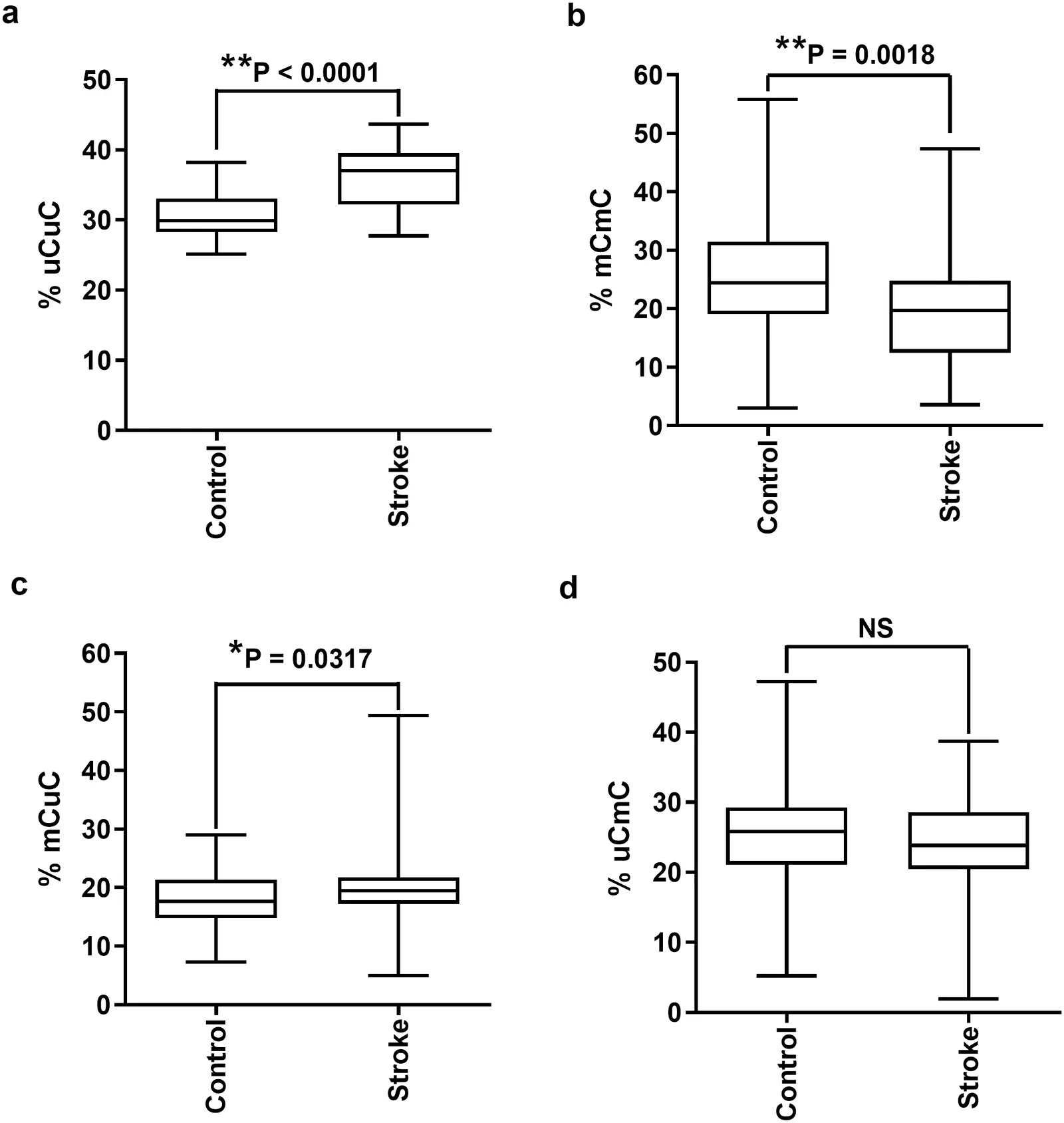
Alu methylation patterns in control and ischemic stroke patients. (a) Fully unmethylated loci (uCuC), (b) fully methylated forms (mCmC), and the two partially methylated patterns, (c) mCuC and (d) uCmC. The boxes represent the interquartile range (25th to 75th percentile), the median lines represent the 50th percentile, and the whiskers represent the minimum and maximum values. All individual data points are displayed. Significance levels: *P < 0.05, **P < 0.01 (Mann-Whitney U test).

### Age correlation and age adjustment

A negative correlation between age and Alu methylation was observed in both normal controls (r = −0.6131, P < 0.0001, Fig 4a) and ischemic stroke patients (r = −0.2834, P = 0.0086, Fig 4b), indicating that older age is associated with decreased Alu methylation levels. To determine whether the significant Alu hypomethylation in ischemic stroke was due to age, we adjusted for age by matching normal samples to the same age as ischemic stroke patients, resulting in 16 age-matched pairs. Even after controlling for age, ischemic stroke patients maintained significantly lower Alu methylation levels compared with controls (P = 0.0182, Fig 4c). In the multivariable logistic regression analysis, Alu methylation remained associated with ischemic stroke status after adjustment for age and sex (adjusted OR = 0.841 per one percentage-point increase in Alu methylation, 95% CI = 0.709–0.979, P = 0.0327; Table 2).

**Fig 4:**
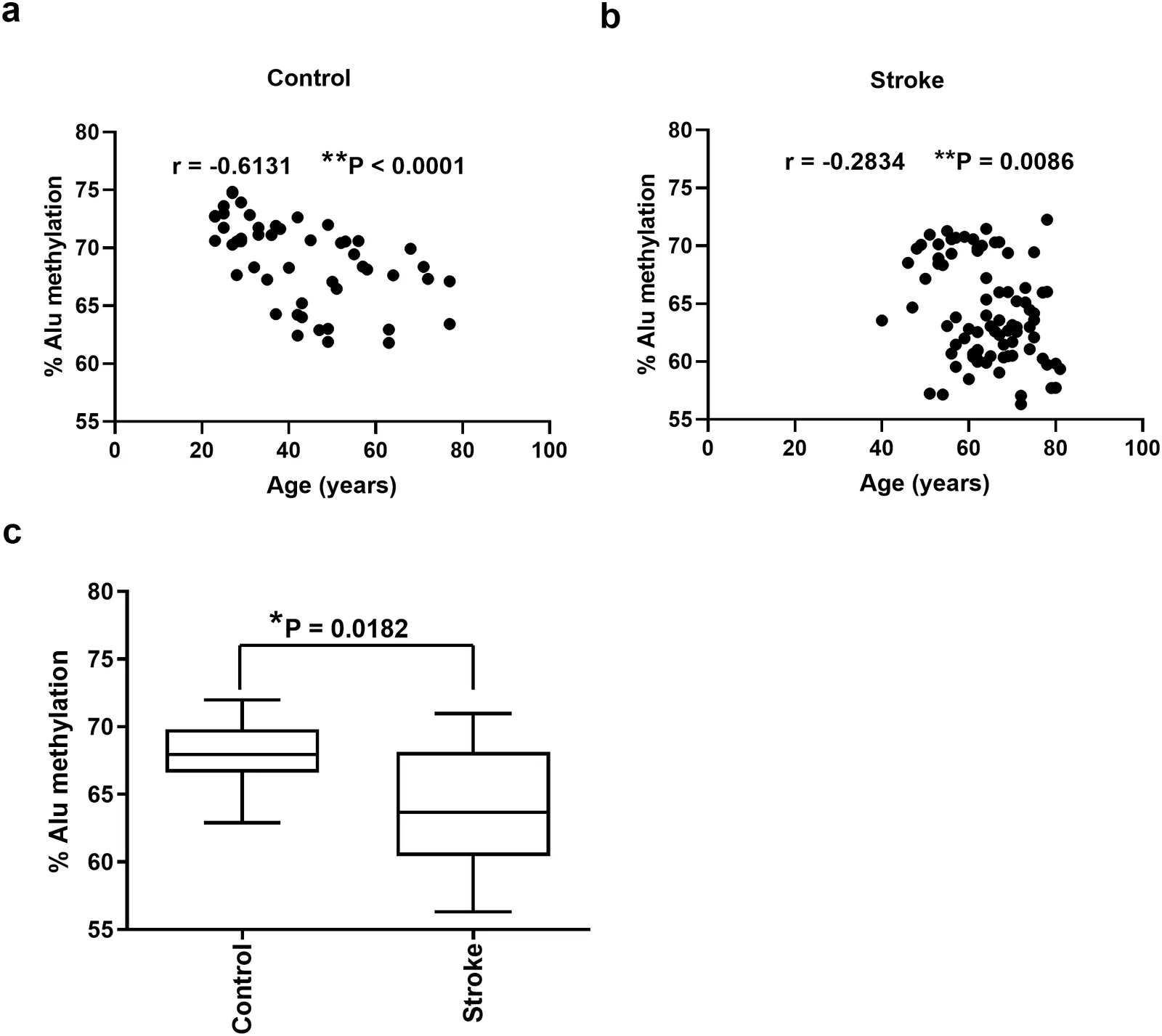
Association between %Alu methylation and age in controls (a) and ischemic stroke patients (b). Spearman’s rank correlation coefficients (r) and P values are shown in panels a and b. (c) Values of independent age-matched pairs each plot represents the levels of %Alu methylation in control and patient. The comparison was performed using the two-sided Wilcoxon matched-pairs signed-rank test. *P < 0.05, **P < 0.01.

**Table 2:** Multivariable logistic regression analysis of factors associated with ischemic stroke status.

Variable	Adjusted OR	95% CI	P value
Alu methylation	0.841	0.709-0.979	0.0327
Age	1.129	1.076-1.197	<0.0001
Male sex	22.05	6.404-99.42	<0.0001

Note: Ischemic stroke status was coded as 0 for controls and 1 for patients with ischemic stroke. Adjusted ORs are reported per one percentage-point increase in Alu methylation and per one-year increase in age. Female sex was used as the reference category.

### Association between Alu methylation and selected clinical characteristics

Disease duration was not significantly correlated with Alu methylation in the unadjusted Spearman analysis (r = −0.1594, P = 0.1450; Fig 5a). However, after adjustment for age and sex, longer disease duration was associated with lower Alu methylation (β = −0.556, 95% CI = −0.994 to −0.117, P = 0.0137; Table 3). In the unadjusted analysis, Alu methylation was lower in patients with a history of multiple stroke events than in those with a single recorded event (P = 0.0323; Fig 5b). However, this association was not statistically significant after adjustment for age and sex (β = −2.271, 95% CI = −4.584 to 0.042, P = 0.0542; Table 3).

**Fig 5:**
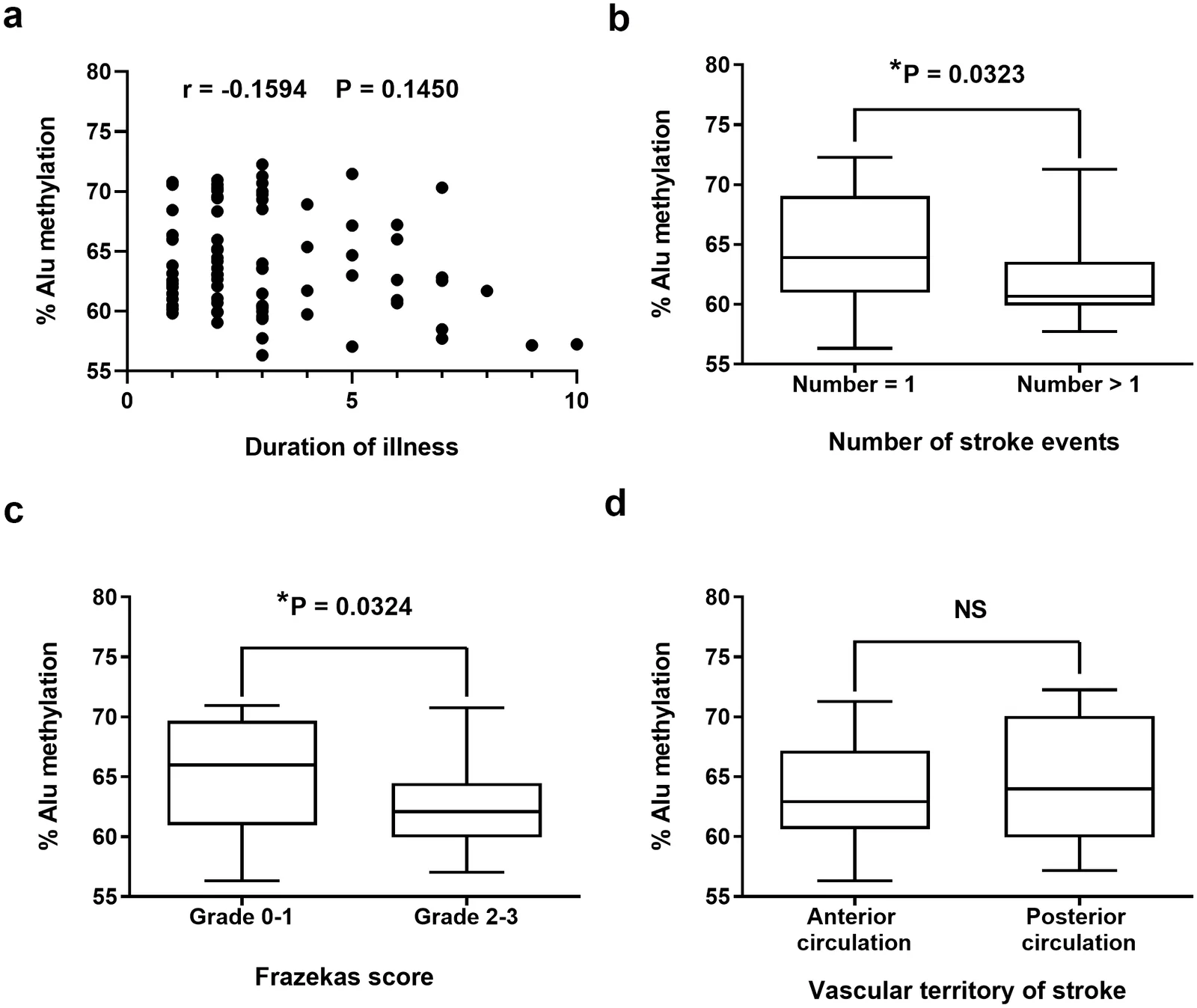
Correlation between Alu methylation and stroke characteristics. (a) Correlation between %Alu methylation levels and duration of illness. (b) %Alu methylation in two subgroups: single stroke event or multiple events. (c) %Alu methylation in two subgroups: stroke with Fazekas score into Grade 0-1 and Grade 2-3 groups. (d) %Alu methylation in two subgroups: anterior and posterior circulation strokes. Spearman’s rank correlation was used in panel a, and the two-sided Mann-Whitney U test was used in panels b-d. The boxes represent the interquartile range (25th to 75th percentile), the median lines represent the 50th percentile, and the whiskers represent the minimum and maximum values. *P < 0.05.

**Table 3:** Associations between Alu methylation and selected clinical characteristics, adjusted for age and sex.

Clinical characteristic	n	Adjusted β	95% CI	P value
Disease duration (per year)	85	−0.556	−0.994 to −0.117	0.0137
Multiple versus single stroke events	85	−2.271	−4.584 to 0.042	0.0542
Fazekas scores 2–3 versus 0–1	56	−2.296	−4.772 to 0.180	0.0684
Posterior versus anterior circulation	81	1.318	−0.977 to 3.613	0.2564

Note: β values were obtained from separate linear regression models adjusted for age and sex. Reference groups were single stroke event, Fazekas scores of 0–1, and anterior circulation stroke. Patients without available Fazekas scores (n = 29) and those with involvement of both circulations (n = 4) were excluded from the respective analyses. All tests were two-sided.

Alu methylation was also lower in patients with Fazekas scores of 2−3 than in those with scores of 0−1 in the unadjusted analysis (P = 0.0324; Fig 5c). After adjustment for age and sex, the association was no longer statistically significant (β = −2.296, 95% CI = −4.772 to 0.180, P = 0.0684; Table 3). Alu methylation did not differ significantly between anterior and posterior circulation strokes before adjustment (P = 0.2201; Fig 5d) or after adjustment for age and sex (β = 1.318, 95% CI = −0.977 to 3.613, P = 0.2564; Table 3).

The residuals in the disease-duration model were not normally distributed (Shapiro-Wilk W = 0.9649, P = 0.0205), whereas no significant deviation from normality was found in the other three models (all P > 0.05). Alu methylation levels did not differ significantly between patients with and without hypertension (n = 67 and 18, respectively; P = 0.2419) or between those with and without diabetes mellitus (n = 30 and 55, respectively; P = 0.1857; S2 Fig).

## Discussion

Our findings revealed a reduction in Alu methylation in ischemic stroke patients compared to healthy controls, we measured Alu repetitive elements within DNA that represent more than 10% of the genome, therefore can represent the overall methylation status in the human genome [18,30,31]. Alu methylation is therefore commonly used as a surrogate measure of global DNA methylation rather than as a marker of a stroke-specific gene [32]. Furthermore, when using COBRA-Alu, ischemic stroke patients had higher percentages of fully unmethylated (uCuC) and partially methylated loci (mCuC), and a lower percentage of fully methylated loci (mCmC). These data suggest that controls may reflect a trend toward hypomethylation that represents a unique pattern in ischemic stroke patients, similar to previous studies that Alu hypomethylation reflects disease states including osteoporosis, hypertension, type 2 diabetes, and cancer, but each has a different specific pattern [29,33–36]. However, within the ischemic stroke group, Alu methylation did not differ significantly between patients with and without hypertension or diabetes mellitus. Because the controls were selected to have no known chronic diseases, we could not determine whether these comorbidities contributed to the difference between the ischemic stroke and control groups.

Age was negatively correlated with Alu methylation in both groups. However, Alu methylation remained lower in patients with ischemic stroke in the age-matched comparison, and the association with stroke status remained significant after adjustment for age and sex. These findings suggest that age is associated with Alu hypomethylation but does not fully explain the lower Alu methylation observed in patients with ischemic stroke.

The relationship between Alu hypomethylation and stroke remains unclear. It is possible that previous studies have established the idea that, during stroke, the brain releases pro-inflammatory cytokines such as TNF-α and IL-6, which generate reactive oxygen species (ROS) [37–41]. The generation of ROS causes oxidative stress, which may lead to increases in 8-oxoguanine (8-oxoG) and elicit a hyperproduction of glutathione to prevent DNA damage [42–44]. This hyperproduction of glutathione binds to the methyl donor S-adenosylmethionine (SAM), which may ultimately decrease the activity of DNA methyltransferases (DNMTs), leading to selective decreases in methylation levels [45–47].

DNA methylation may play a protective role for DNA via association with youth-DNA-gap complex. Previous studies have shown that the release of HMGB1 results in a decrease in the number of DNA gaps produced by HMGB1 and contributes to the accumulation of DNA damage, ultimately leading to a reduction in Alu methylation. These epigenetic changes can initiate cellular senescence and represent an upstream molecular event in the development of non-communicable diseases [28,48–53]. Therefore, Alu hypomethylation might promote endothelial dysfunction, vascular inflammation, and disruption of atherogenic and coagulation pathways, potentially inducing stroke [54,55]. From this perspective, the hypomethylation of Alu elements demonstrated across stroke patients suggests a bidirectional relationship whereby stroke increases the oxidative stress response and exacerbates hypomethylation, and hypomethylation also increases genomic instability that contributes to the risk of a stroke.

Disease duration was not significantly associated with Alu methylation in the unadjusted analysis, but an association became apparent after adjustment for age and sex. Longer disease duration was associated with lower Alu methylation in the adjusted model. This difference may reflect the effect of adjusting for age. In the unadjusted comparisons, methylation levels were lower in patients with a history of multiple stroke events and in those with Fazekas scores of 2–3. Neither association was statistically significant after adjustment for age and sex. The Fazekas score reflects the burden of white matter hyperintensities rather than overall clinical stroke severity [56–58]. Alu methylation did not differ significantly between anterior and posterior circulation strokes, either before or after adjustment. These cross-sectional findings do not show that Alu methylation predicts future stroke recurrence or prognosis.

However, this study has several limitations. It was a single-center cross-sectional study with a relatively small sample. The control and stroke groups differed in age and sex, and only 16 age-matched pairs were available. Despite age matching and multivariable adjustment, residual confounding remains possible. Residuals in the disease-duration model were non-normal, so this association should be interpreted cautiously. Peripheral-blood Alu methylation was used as a surrogate for global DNA methylation and does not reflect stroke-specific gene or brain-tissue changes. Inflammatory biomarkers were not measured at the time of blood collection. Finally, the cross-sectional design does not allow conclusions about causality, changes over time, or prognostic value. Larger multicenter longitudinal studies are needed to confirm these findings.

## Conclusions

Alu methylation was lower in patients with ischemic stroke than in healthy controls and remained associated with ischemic stroke status after adjustment for age and sex. Within the stroke group, longer disease duration was associated with lower Alu methylation after adjustment, whereas the associations with multiple stroke events, higher Fazekas scores, and stroke circulation location were not statistically significant. These findings do not establish Alu methylation as a stroke-specific biomarker or support its use for prediction or prognosis. Longitudinal studies in independent populations are needed to assess its clinical relevance.

## Supporting information

S1 Fig Representative polyacrylamide gel image of COBRA-Alu digestion products. TaqI- and TasI-digested PCR products were separated on an 8% gel. Expected fragments were 133, 90, 75, 58, and 43 bp. C1 and S1 represent a healthy control and an ischemic stroke sample, respectively, each loaded in two adjacent lanes. Ladder, DNA size marker. The original uncropped and unadjusted image is provided in S1 Raw images.
(TIF)

S2 Fig Alu methylation levels according to hypertension and diabetes mellitus status among patients with ischemic stroke. (a) Alu methylation levels in patients with and without hypertension (P = 0.2419). (b) Alu methylation levels in patients with and without diabetes mellitus (P = 0.1857). Differences were assessed using the two-sided Mann-Whitney U test. Boxes represent the median and interquartile range, and whiskers indicate the minimum and maximum values.
(TIF)

S1 Data De-identified individual-level data and variable definitions. Direct participant identifiers and age data were removed to protect participant privacy.
(XLSX)

S1 Raw images Original uncropped and unadjusted gel image underlying S1 Fig. From left to right, the lanes show the DNA ladder, two adjacent lanes for C1, and two adjacent lanes for S1. C1 represents a healthy control sample, and S1 represents an ischemic stroke sample. The gel image was acquired in fluorescence mode using a Typhoon FLA 7000 biomolecular imager. All lanes are shown, and none were omitted.
(PDF)

## Abbreviations

COBRA: Combined Bisulfite Restriction Analysis
DNMT: DNA methyltransferase
HMGB1: High mobility group box 1
IL6: Interleukin-6
ROS: Reactive oxygen species
SAM: S-adenosylmethionine
TNF: Tumor necrosis factor

## References

[1] Yadav VK, Gupta R, Assiri AA, Uddin J, Ishaqui AA, Kumar P, et al. Role of nanotechnology in ischemic stroke: Advancements in targeted therapies and diagnostics for enhanced clinical outcomes. J Funct Biomater. 2025;16(1):8. doi: 10.3390/jfb16010008 39852564 PMC11766075

[2] Fu X, Li J, Yang S, Jing J, Zheng Q, Zhang T, et al. Blood-brain barrier repair: Potential and challenges of stem cells and exosomes in stroke treatment. Front Cell Neurosci. 2025;19:1536028. doi: 10.3389/fncel.2025.1536028 40260076 PMC12009835

[3] Murphy SJX, Werring DJ. Stroke: Causes and clinical features. Medicine. 2023;51(9):602–7. doi: 10.1016/j.mpmed.2023.06.003 PMC7409792 32837228

[4] Feigin VL, Brainin M, Norrving B, Martins SO, Pandian J, Lindsay P. World stroke organization: Global stroke fact sheet 2025. Int J Stroke. 2025;20(2):132–44. 10.1177/17474930241308142 PMC11786524 39635884

[5] Kalailingam P, Rannikmae K, Hausman-Kedem M, Musolino PL, Ruigrok YM. Genetic insights into hemorrhagic stroke and vascular malformations: Pathogenesis and emerging therapeutic strategies. Stroke. 2025;56(5):1298–311. doi: 10.1161/STROKEAHA.124.045182 40084704 PMC12037314

[6] Jagodic A, Zivalj D, Krsek A, Baticic L. Genetic architecture of ischemic stroke: Insights from genome-wide association studies and beyond. J Cardiovasc Dev Dis. 2025;12(8):281. doi: 10.3390/jcdd12080281 40863347 PMC12386999

[7] Yang T, Feng L. Research progress of epigenetic regulation in stroke treatment. Life Conflux. 2025;1(2):e124. doi: 10.71321/y2sx8j49

[8] Gallego-Fabrega C, Cullell N, Fernández-Cadenas I. How epigenetics impacts stroke risk and outcomes through DNA methylation: A systematic review. J Cereb Blood Flow Metab. 2025;46(6):1369–85. doi: 10.1177/0271678x251322032 PMC11866340 40012472

[9] Stanzione R, Cotugno M, Bianchi F, Marchitti S, Forte M, Volpe M, et al. Pathogenesis of ischemic stroke: Role of epigenetic mechanisms. Genes (Basel). 2020;11(1):89. doi: 10.3390/genes11010089 31941075 PMC7017187

[10] Arruri V, Joshi P, Vemuganti R. DNA methylation and hydroxymethylation dynamics in the aging brain and its impact on ischemic stroke. Neurochem Int. 2025;188:106007. doi: 10.1016/j.neuint.2025.106007 40513956 PMC12331423

[11] Baccarelli A, Wright R, Bollati V, Litonjua A, Zanobetti A, Tarantini L, et al. Ischemic heart disease and stroke in relation to blood DNA methylation. Epidemiology. 2010;21(6):819–28. doi: 10.1097/EDE.0b013e3181f20457 20805753 PMC3690659

[12] Wang Y, Zhang L, Lyu T, Cui L, Zhao S, Wang X, et al. Association of DNA methylation/demethylation with the functional outcome of stroke in a hyperinflammatory state. Neural Regen Res. 2024;19(10):2229–39. doi: 10.4103/1673-5374.392890 38488557 PMC11034580

[13] Xie J, Xie L, Wei H, Li X-J, Lin L. Dynamic regulation of DNA methylation and brain functions. Biology (Basel). 2023;12(2):152. doi: 10.3390/biology12020152 36829430 PMC9952911

[14] Haider R, Ahmed Z, Zameer S. DNA and its functional significance in health and disease. J Med Sci. 2025;3(3):1–3.

[15] Ur Rehman S, Abdullah M, Khan ZK, Shurovi M. The role of DNA methylation and histone modifications in the pathogenesis of hematological malignancies and solid cancers: Mechanisms, clinical implications, and therapeutic potential. Asian J Med Biol Res. 2025;11(2):23–36. doi: 10.3329/ajmbr.v11i2.79668

[16] Dhar GA, Saha S, Mitra P, Nag Chaudhuri R. DNA methylation and regulation of gene expression: Guardian of our health. Nucleus (Calcutta). 2021;64(3):259–70. doi: 10.1007/s13237-021-00367-y 34421129 PMC8366481

[17] Gwee J, Tian W, Qian S, Zhong X. DNA methylation dynamics: Patterns, regulation, and function. Curr Opin Plant Biol. 2025;88:102787. doi: 10.1016/j.pbi.2025.102787 40972239 PMC13054798

[18] Xing J, Hedges DJ, Han K, Wang H, Cordaux R, Batzer MA. Alu element mutation spectra: Molecular clocks and the effect of DNA methylation. J Mol Biol. 2004;344(3):675–82. doi: 10.1016/j.jmb.2004.09.058 15533437

[19] Mutirangura A. DNA damage, epigenetics, and aging. Epigenetics and DNA Damage. Elsevier: 2022. 139–56. doi: 10.1016/b978-0-323-91081-1.00010-8

[20] Charoenvicha C, Thongsroy J, Apaijai N, Attachaipanich T, Sirimaharaj W, Khwanngern K, et al. Alterations of senescence-associated markers in patients with non-syndromic cleft lip and palate. Sci Rep. 2024;14(1):22555. doi: 10.1038/s41598-024-74353-0 39343816 PMC11439953

[21] Jiraboonsri S, Hemvipat P, Kamolratanakul S, Bhummaphan N, Siritientong T, Kitkumthorn N, et al. CpG methylation changes in Alu repetitive sequences in normal aging due to diastolic hypertension in human dermal fibroblasts from the facial area. Biomed Rep. 2023;20(1):5. doi: 10.3892/br.2023.1693 38222864 PMC10784876

[22] Kousar T, Rahim MSM, Iqbal S, Yousaf F, Sanaullah M. Applications of deep learning algorithms in ischemic stroke detection, segmentation, and classification. Artif Intell Rev. 2025;58(5). doi: 10.1007/s10462-025-11119-8

[23] Yaghi S, Johansen MC, Ghannam M, Kasner SE, Singh N, Sposato LA. Proposal for the ischemic stroke phenotyping system 2025: ISPS25. Stroke. 2025. 10.1161/STROKEAHA.125.051169 PMC12614268 41121616

[24] Shahid MI, Khalid M, Sultan U, Zainullah, Jamil H, Zafar Q. Exploring the pattern of stroke in young adults: clinical presentations, risk factors, and implications for early detection and management. Indus J Biosci Res. 2025;3(5):835–8. doi: 10.70749/ijbr.v3i5.1561

[25] Vongprommool A, Mutirangura A, Pavasant P, Subbalekha K. Alu methylation level, morphological, and senescence changes during in vitro aging of human dental pulp stem cells. Tissue Cell. 2024;90:102512. doi: 10.1016/j.tice.2024.102512 39126831

[26] Meevassana J, Serirodom S, Prabsattru P, Boonsongserm P, Kamolratanakul S, Siritientong T, et al. Alu repetitive sequence CpG methylation changes in burn scars. Burns. 2022;48(6):1417–24. doi: 10.1016/j.burns.2021.10.002 34657766

[27] Thongsroy J, Mutirangura A. The association between Alu hypomethylation and the severity of hypertension. PLoS One. 2022;17(7):e0270004. doi: 10.1371/journal.pone.0270004 35802708 PMC9269909

[28] Thongsroy J, Patchsung M, Mutirangura A. The association between Alu hypomethylation and severity of type 2 diabetes mellitus. Clin Epigenetics. 2017;9:93. doi: 10.1186/s13148-017-0395-6 28883893 PMC5580285

[29] Thongsroy J, Mutirangura A. Decreased Alu methylation in type 2 diabetes mellitus patients increases HbA1c levels. J Clin Lab Anal. 2023;37(17–18):e24966. doi: 10.1002/jcla.24966 37743692 PMC10623537

[30] Ade C, Roy-Engel AM, Deininger PL. Alu elements: An intrinsic source of human genome instability. Curr Opin Virol. 2013;3(6):639–45. doi: 10.1016/j.coviro.2013.09.002 24080407 PMC3982648

[31] Kim S, Cho C-S, Han K, Lee J. Structural variation of alu element and human disease. Genomics Inform. 2016;14(3):70–7. doi: 10.5808/GI.2016.14.3.70 27729835 PMC5056899

[32] Yang AS, Estécio MRH, Doshi K, Kondo Y, Tajara EH, Issa J-PJ. A simple method for estimating global DNA methylation using bisulfite PCR of repetitive DNA elements. Nucleic Acids Res. 2004;32(3):e38. doi: 10.1093/nar/gnh032 14973332 PMC373427

[33] Tiwawech D, Srisuttee R, Rattanatanyong P, Puttipanyalears C, Kitkumthorn N, Mutirangura A. Alu methylation in serum from patients with nasopharyngeal carcinoma. Asian Pac J Cancer Prev. 2014;15(22):9797–800. doi: 10.7314/apjcp.2014.15.22.9797 25520107

[34] Puttipanyalears C, Subbalekha K, Mutirangura A, Kitkumthorn N. Alu hypomethylation in smoke-exposed epithelia and oral squamous carcinoma. Asian Pac J Cancer Prev. 2013;14(9):5495–501. doi: 10.7314/apjcp.2013.14.9.5495 24175848

[35] Woraruthai T, Charoenlap C, Hongsaprabhas C, Mutirangura A, Honsawek S. Alu hypermethylation and high oxidative stress in patients with musculoskeletal tumors. PeerJ. 2018;6:e5492. doi: 10.7717/peerj.5492 30128216 PMC6098941

[36] Kitkumthorn N, Keelawat S, Rattanatanyong P, Mutirangura A. LINE-1 and Alu methylation patterns in lymph node metastases of head and neck cancers. Asian Pac J Cancer Prev. 2012;13(9):4469–75. doi: 10.7314/apjcp.2012.13.9.4469 23167363

[37] Pawluk H, Woźniak A, Tafelska-Kaczmarek A, Kosinska A, Pawluk M, Sergot K, et al. The role of IL-6 in ischemic stroke. Biomolecules. 2025;15(4):470. doi: 10.3390/biom15040470 40305179 PMC12024898

[38] Iordache MP, Buliman A, Costea-Firan C, Gligore TCI, Cazacu IS, Stoian M, et al. Immunological and inflammatory biomarkers in the prognosis, prevention, and treatment of ischemic stroke: A review of a decade of advancement. Int J Mol Sci. 2025;26(16):7928. doi: 10.3390/ijms26167928 40869247 PMC12386479

[39] Xue Y, Zeng X, Tu W-J, Zhao J. Tumor necrosis factor-α: The next marker of stroke. Dis Markers. 2022;2022:2395269. doi: 10.1155/2022/2395269 35265224 PMC8898850

[40] Fanzhou R, Yuding L, Pingchuan L, Hai X, Xu L, Jian W. The role of TNF signaling pathway in post-stroke cognitive impairment: A systematic review. Ann Med. 2025;57(1):2543519. doi: 10.1080/07853890.2025.2543519 40781737 PMC12337740

[41] Xiao L, Huang Y, Wu L, Zeng S, Qiu C, Li X, et al. The role of inflammation in Ischemic stroke: from biomarker to treatment. Front Immunol. 2025;16:1608353. doi: 10.3389/fimmu.2025.1608353 41080593 PMC12510868

[42] Uddin T. Oxidative genomic or genotoxic stress in neurodegeneration: Mechanisms and therapeutic avenues. AIMS Neurosci. 2025;12(3):369–90. doi: 10.3934/Neuroscience.2025020 41103972 PMC12521933

[43] Panda S, Chatterjee O, Mukherjee G, Chatterjee S. Human diseases induced by oxidative damage in DNA. Nucleic acid biology and its application in human diseases. Springer Nature Singapore; 2023. 135–66. doi: 10.1007/978-981-19-8520-1_5

[44] Zhang W, Zhong R, Qu X, Xiang Y, Ji M. Effect of 8-hydroxyguanine DNA glycosylase 1 on the function of immune cells. Antioxidants (Basel). 2023;12(6):1300. doi: 10.3390/antiox12061300 37372030 PMC10295362

[45] Lee YH, Ren D, Jeon B, Liu H w. S-Adenosylmethionine: more than just a methyl donor. Nat Prod Rep. 2023;40(9):1521–49. 10.1039/d2np00086e PMC10491745 36891755

[46] Abdelraheem E, Thair B, Varela RF, Jockmann E, Popadić D, Hailes HC, et al. Methyltransferases: Functions and applications. Chembiochem. 2022;23(18):e202200212. doi: 10.1002/cbic.202200212 35691829 PMC9539859

[47] Fernández-Ramos D, Lopitz-Otsoa F, Lu SC, Mato JM. S-Adenosylmethionine: A multifaceted regulator in cancer pathogenesis and therapy. Cancers (Basel). 2025;17(3):535. doi: 10.3390/cancers17030535 39941901 PMC11816870

[48] Yasom S, Watcharanurak P, Bhummaphan N, Thongsroy J, Puttipanyalears C, Settayanon S, et al. The roles of HMGB1-produced DNA gaps in DNA protection and aging biomarker reversal. FASEB Bioadv. 2022;4(6):408–34. doi: 10.1096/fba.2021-00131 35664831 PMC9164245

[49] Patchsung M, Settayanon S, Pongpanich M, Mutirangura D, Jintarith P, Mutirangura A. Alu siRNA to increase Alu element methylation and prevent DNA damage. Epigenomics. 2018;10(2):175–85. doi: 10.2217/epi-2017-0096 29336607

[50] Watcharanurak P, Mutirangura A. Genome wide hypomethylation and youth-associated DNA gap reduction promoting DNA damage and senescence-associated pathogenesis. MRAJ. 2023;11(12). doi: 10.18103/mra.v11i12.4952

[51] Watcharanurak P, Mutirangura A. DNA protection epigenetic marks: “Youth-associated genomic stabilization DNA gaps” (youth-DNA-gaps). ScienceAsia. 2025;51S(1):1. doi: 10.2306/scienceasia1513-1874.2025.s005

[52] Watcharanurak P, Mutirangura A. Human RNA-directed DNA methylation methylates high-mobility group box 1 protein-produced DNA gaps. Epigenomics. 2022;14(12):741–56. doi: 10.2217/epi-2022-0022 35762252

[53] Thongsroy J, Mutirangura A. The inverse association between DNA gaps and HbA1c levels in type 2 diabetes mellitus. Sci Rep. 2023;13(1):18987. doi: 10.1038/s41598-023-46431-2 37923892 PMC10624909

[54] Ayomide OT, Chavda V, Chaurasia B, Bassey E, Dang K, Oyoyo HD, et al. Current advancement and patient outcomes in reperfusion brain injuries after stroke: A comparative analysis of thrombolysis and thrombectomy. Brain Behav. 2025;15(8):e70705. doi: 10.1002/brb3.70705 40760789 PMC12321978

[55] Liang J, Yang F, Li Z, Li Q. Epigenetic regulation of the inflammatory response in stroke. Neural Regen Res. 2025;20(11):3045–62. doi: 10.4103/NRR.NRR-D-24-00672 39589183 PMC11881735

[56] Caruso P, Liccari M, Prandin G, Vinci P, Pellicori F, Fiotti N, et al. Lipoprotein-a and white matter abnormalities: Predicting small vessel disease in young patients with ischemic cerebrovascular events. J Neurol. 2025;272(6):379. doi: 10.1007/s00415-025-13111-2 40327115 PMC12055645

[57] Zerna C, Yu AYX, Modi J, Patel SK, Coulter JI, Smith EE, et al. Association of white matter hyperintensities with short-term outcomes in patients with minor cerebrovascular events. Stroke. 2018;49(4):919–23. doi: 10.1161/STROKEAHA.117.017429 29540612

[58] Hairus S MN, Srinivasan A, Shaik Mohideen SA, MacAlevey NCYL, Chan MY, Leow AST, et al. Cerebral small-vessel disease markers on magnetic resonance imaging as predictors of recurrent vascular events and death in ischemic stroke: A systematic review and meta-analysis. J Am Heart Assoc. 2025;14(17):e040082. doi: 10.1161/JAHA.124.040082 40847482 PMC12553415

