# Supplementary material for: Alu hypomethylation in ischemic stroke: Associations with selected clinical characteristics
Source: PLoS One. 2026 Oct 21;21(10):e0360371. doi: 10.1371/journal.pone.0360371 (PMC13686516; doi:10.1371/journal.pone.0360371)
Supplement: Supplementary file 1 [file pone.0360371.s004.pdf]

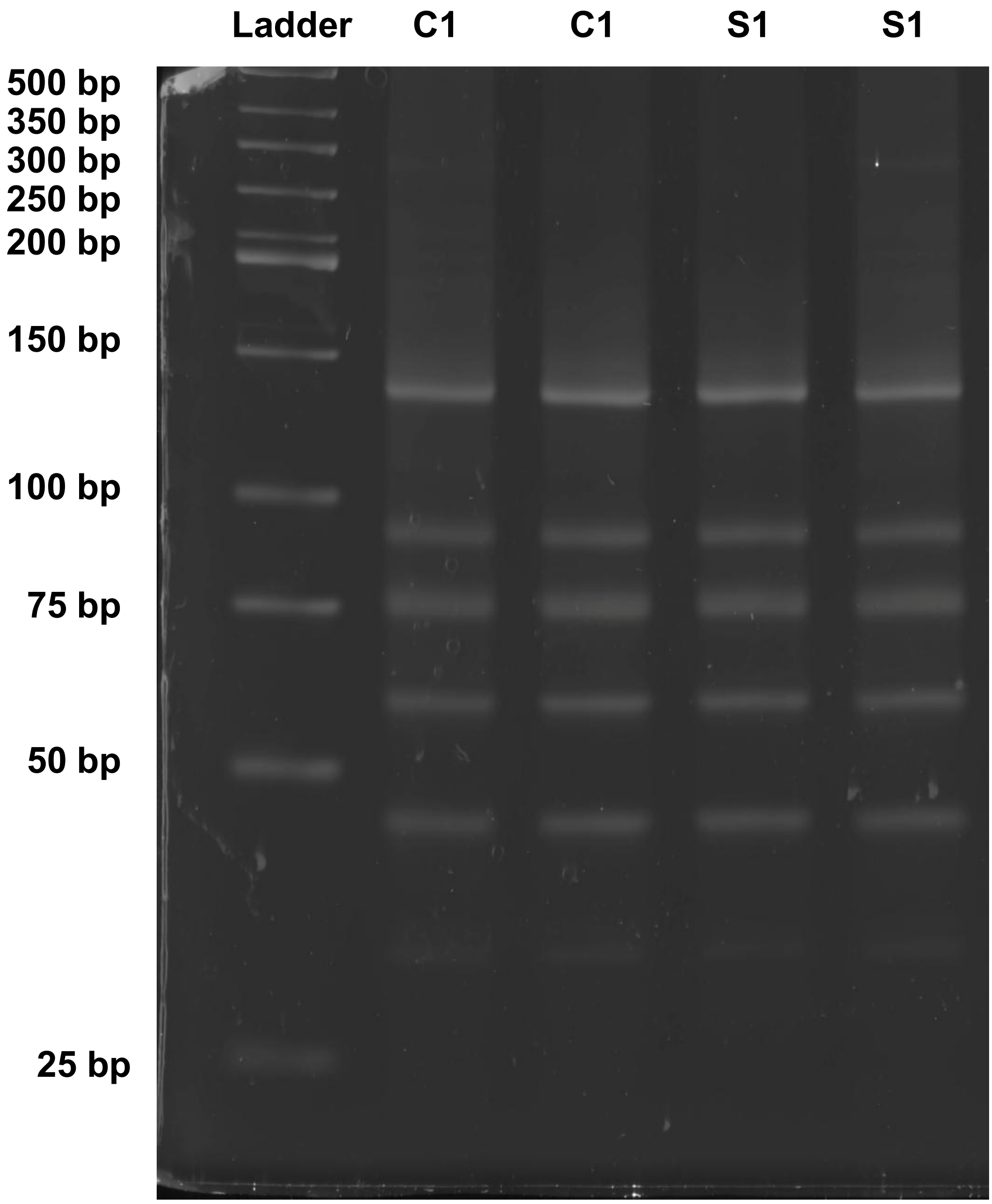

S1 Raw images. Original uncropped and unadjusted gel image underlying S1 Fig. From left to right, the lanes show the DNA ladder, two adjacent lanes for C1, and two adjacent lanes for S1. C1 represents a healthy control sample, and S1 represents an ischemic stroke sample. The gel image was acquired in fluorescence mode using a Typhoon FLA 7000 biomolecular imager. All lanes are shown, and none were omitted.
